# Expression of the Axonal Membrane Glycoprotein M6a Is Regulated by Chronic Stress

**DOI:** 10.1371/journal.pone.0003659

**Published:** 2009-01-30

**Authors:** Ben Cooper, Eberhard Fuchs, Gabriele Flügge

**Affiliations:** 1 Clinical Neurobiology Laboratory, German Primate Center, Leibniz Institute for Primate Research, Göttingen, Germany; 2 Department of Neurology, Medical School, University of Göttingen, Göttingen, Germany; 3 DFG Research Center Molecular Physiology of the Brain (CMPB), University of Göttingen, Göttingen, Germany; James Cook University, Australia

## Abstract

It has been repeatedly shown that chronic stress changes dendrites, spines and modulates expression of synaptic molecules. These effects all may impair information transfer between neurons. The present study shows that chronic stress also regulates expression of M6a, a glycoprotein which is localised in axonal membranes. We have previously demonstrated that M6a is a component of glutamatergic axons. The present data reveal that it is the splice variant M6a-Ib, not M6a-Ia, which is strongly expressed in the brain. Chronic stress in male rats (3 weeks daily restraint) has regional effects: quantitative in situ hybridization demonstrated that M6a-Ib mRNA in dentate gyrus granule neurons and in CA3 pyramidal neurons is downregulated, whereas M6a-Ib mRNA in the medial prefrontal cortex is upregulated by chronic stress. This is the first study showing that expression of an axonal membrane molecule is differentially affected by stress in a region-dependent manner. Therefore, one may speculate that diminished expression of the glycoprotein in the hippocampus leads to altered output in the corresponding cortical projection areas. Enhanced M6a-Ib expression in the medial prefrontal cortex (in areas prelimbic and infralimbic cortex) might be interpreted as a compensatory mechanism in response to changes in axonal projections from the hippocampus. Our findings provide evidence that in addition to alterations in dendrites and spines chronic stress also changes the integrity of axons and may thus impair information transfer even between distant brain regions.

## Introduction

The membrane glycoprotein M6a is the only member of the proteolipid protein family of tetraspan proteins to be expressed exclusively by neurons in the central nervous system [1], [2]. Non-neuronal expression of M6a in peripheral tissues is restricted to the apical membranes of polarized epithelial cells within the choroid plexus and proximal renal tubules [3]. Neuronal M6a was formerly suspected to play a role in the formation of nerve cell processes since in cultured cerebellar neurons treated with monoclonal M6a antibody, neurite formation was severely impaired [4]. Moreover, targeted depletion of endogenous M6a expression with small inhibitory RNA (siRNA) attenuated neurite outgrowth and impaired synapse formation [5]. On the other hand, overexpression of M6a in cultured primary hippocampal neurons promoted neurite outgrowth and the formation of filopodial protrusions [5]. However, in a previous publication we showed that the membrane glycoprotein is not present in dendrites, but only in axons of glutamatergic neurons [6]. In the present study, we analyzed the relative abundance of M6a splice variants Ia and Ib in the rat brain and their regulation by chronic stress exposure.

M6a initially attracted attention as a gene downregulated by stress in the hippocampal formation [7], [8]. In humans, chronic stress-induced perturbations of the central nervous system including structural changes in neurons have the potential to lead to psychopathologies [9], [10]. Stress-induced changes in the expression of M6a, a structural protein of axonal membranes, are therefore of particular interest. Stress-induced downregulation of hippocampal M6a has been confirmed in several species using quantitative real-time RT-PCR, a method that allows quantification of mRNA expression levels in homogenates from defined brain regions [8], [11]. In the present study, using in situ hybridization with emulsion autoradiography, we quantified M6a mRNA levels after chronic stress in neurons from distinct hippocampal subregions. Silver grains representing M6a mRNA transcripts were counted in dentate gyrus granule neurons, the cells that extend mossy fiber projections to the hippocampal region CA3. Moreover, we analyzed M6a mRNA expression in the CA3 pyramidal neurons of stressed rats and controls. To induce stress, male rats were submitted to three weeks of daily restraint (6 hr/day) according to established protocols [12], [13].

In addition to the hippocampal pyramidal neurons that respond to chronic stress by retracting their dendrites [14], [15] pyramidal neurons in the medial prefrontal cortex (mPFC) are also sensitive to stress [16]–[18]. Chronic restraint stress in male rats reduced the length of apical dendrites of layer III pyramidal neurons in the right prelimbic cortex (PL) and eliminated inter-hemispheric differences in dendritic length in PL and infralimbic cortex (IL), both of which represent sub-areas of the mPFC [19], [20]. In the present study we quantified M6a mRNA expression in cells of the three mPFC sub-areas, PL, IL and anterior cingulated cortex (ACx) to detect whether chronic stress might also have an effect on axons of prefrontocortical neurons.

## Materials and Methods

### Animals

Adult male Sprague Dawley rats (Harlan-Winkelmann, Borchen, Germany) weighing 250–300 g on arrival were housed in groups of three animals per cage with food and water ad libitum in temperature-controlled rooms (21±1°C) under an inverse light cycle (lights off at 07:00, lights on at 19:00). All handling procedures including stress exposure were performed in the morning under dim red light (see below). Animal experiments were performed in accordance with the European Communities Council Directive of November 24, 1986 (86/EEC) and the US National Institutes of Health Guide for the Care and Use of Laboratory Animals, and were approved by the Lower Saxony Federal State Office for Consumer Protection and Food Safety, Germany.

### Quantitative Real-time RT-PCR

**Figure 1:**
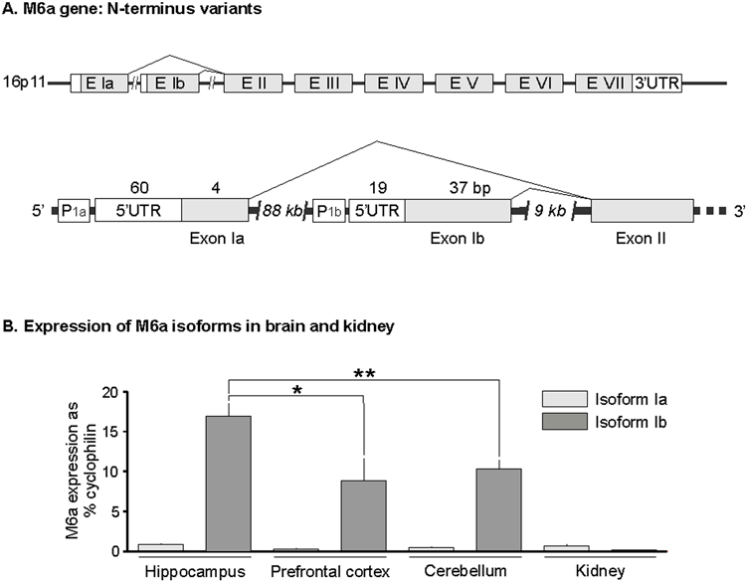
Expression of M6a isoforms Ia and Ib in rat brain and kidney. A: The M6a gene comprises 7 exons (E I to E VII). Because of alternate transcription start sites, two N-terminus variants of M6a transcripts are generated with sequences corresponding to either exon Ia (E Ia) which encodes a short N-terminal domain, or exon Ib (E Ib) which encodes a longer N-terminal domain. B: Constitutive expression of M6a isoforms Ia and Ib in rat brain regions and kidney. For each sample, M6a mRNA transcripts were quantified by RT-PCR and expressed as a percentage of mRNA for the internal reference gene cyclophilin. Data are mean±SEM (standard error of the mean) from n = 9 animals. Statistical differences according to Student's t-test: *, p<0.05; **, p>0.01. UTR, untranslated region.

Cloning of rat M6a cDNA has been previously described [6]. To isolate RNA for RT-PCR, animals were decapitated and brains quickly dissected. Hippocampal formation, prefrontal cortex and cerebellum were dissected and kidneys were also sampled. Total RNA was immediately isolated from the individual tissue samples using the Trizol method (Life Technologies, Rockville, MD, USA) according to the manufacturer's instructions with some modifications. Modifications improving the yield of isolated RNA included a 30 sec sonification step and the addition of linear acrylamide (5 µg/ml) to Trizol homogenates. DNase I digestion was performed and total RNA was purified using phenol/isoamyl/chloroform and subsequent isopropyl/sodium acetate precipitation [21]. The integrity and quantity of purified RNA was assessed by spectrophotometry and subsequently confirmed with RNA 6000 Nano Labchip technology (Agilent Technologies Sales, Waldbronn, Germany). Complementary DNA (cDNA) was synthesized from mRNA transcripts using oligo (dT)_12–18_ primers and Superscript II reverse transcriptase (Invitrogen, Karlsruhe, Germany) according to manufacturers' instructions. Primer Express software v2.0 (Applied Biosystems; Darmstadt, Germany) was used to design gene-specific primers with amplicons ranging between 50–150 bp in length. The intron-exon organisation of murine M6a and M6b genes has been previously described [2]. M6a isoform Ia encodes a short N-terminal domain, whereas M6a isoform Ib encodes a longer N-terminal domain containing a putative PKC phosphorylation site. In the present study rat ESTs corresponding to M6a isoforms Ia (Genebank Acc: DV216104) and Ib (Genebank Acc: CO401660) were identified in the NCBI database and intron-exon boundaries were mapped according to genomic rat DNA (Contig Accession: NW_001084718). Thus, three types of primers were synthesized for real-time RT-PCR analysis; i) primers recognizing the 3′-UTR region of M6a (common to all isoforms of M6a); ii) primers specific for M6a isoform Ia; and iii) primers specific for M6a isoform Ib (Table 1; Fig. 1).

**Table 1:** Primer used for quantitative RT-PCR.

Primer Pairs	Forwards	Reverse
**M6a 3′UTR**	5′-TTCAACGTGTGGACCATCTGC	5′-AGAGATTTGCTCCCTCCACGAG
**M6a Isoform Ia**	5′-GCCTGCCTGGTCTTTACACTTC	5′-CACTCAAAACACCCCATATCCA
**M6a Isoform Ib**	5′-CCTGAAGAAAGGTAGCCATGGA	5′-GCAGCACTCAAAACACCCTTTT
**Cyclophilin**	5′-CAAATGCTGGACCCAACACA	5′-TGCCATCCAACCACTCAGTCT

A quantitative analysis of gene expression was performed using the 7500 Real-time PCR (Applied Biosystems, Darmstadt, Germany) in combination with Quantitect SYBR green technology (Qiagen, Hilden, Germany). The light cycler was programmed to the following conditions: an initial PCR activation step of 10 min at 95°C, followed by cycling steps; denaturation for 15 sec at 95°C, annealing for 30 sec at 60°C, and elongation for 60 sec at 72°C; these steps were repeated for 40 cycles. Details of the quantitative RT-Real time PCR have been described before [21]. Dissociation curves were generated for all PCR products to confirm that SYBR green emission is detected from a single PCR product [22]. The relative abundance of M6a mRNA transcripts was calculated in reference to the mRNA levels of the internal reference gene cyclophilin as described before [8].

### In situ hybridization

Fresh frozen brains from adult rats were cut on a cryostat and 10 µm cryosections were thaw-mounted on gelatine-coated slides. Sections were dried at room temperature for 20 min, fixed in 4% buffered paraformaldehyde (PFA, pH 7.2), rinsed in phosphate-buffered saline (PBS; 0.1 mM phosphate buffer, 0.9% NaCl, pH 7.2), dehydrated through graded alcohols, air dried and frozen at −80°C. Prior to hybridization, sections were rehydrated through graded alcohols, fixed in 4% PFA, washed in PBS, acetylated (0.1 M triethanolamine, 0.25% acetic anhydride), washed in PBS and dehydrated once again through graded alcohols. M6a plasmid DNA [6] was linearized and riboprobes were synthesized with T7 and SP6 RNA polymerases (Promega, Madison, WI, USA) for the antisense and sense probe, respectively, in the presence of 9.25 MBq of ^33^P-UTP (ICN; specific activity 3000 Ci/mmol) for 1 h at 37°C. Probes were purified with Microspin S-400 HR columns (Amersham Pharmacia, Freiburg, Germany) and hybridization buffer (50% deionised formamide, 10% dextran sulphate, 0.3 M NaCl, 1 mM EDTA, 10 mM Tris-HCl, ph 8.0, 500 µg/ml tRNA, 0.1 M dithiothreitol, and 1× Denhardt's solution) was added to give a final probe activity of 5×10^4^ CPM. The hybridization mixture containing the probe was denatured at 70°C for 10 min, cooled to 55°C, and pipetted directly onto sections (80 µl/section). Hybridization was performed for 18 hrs at 43°C. Sections were subsequently washed in 4× SSC (0.6 M NaCl, 0.06 M citric acid), 2× SSC, and 0.5× SSC for 10 min each at 37°C. Following 1 hr incubation at 70°C in 0.2× SSC, sections were washed twice in 0.1× SSC, once at 37°C and again at room temperature, for 10 min each. Sections were dehydrated through graded alcohols, air dried, and exposed to Bio-Max MR film (Amersham Pharmacia, Freiburg, Germany) for 4 days at 4°C. Films were developed and fixed with GBX (Kodak, Rochester, NJ, USA).

### Quantitative in situ hybridization

Rat brains were prepared for cryosectioning under RNAse-free conditions as previously described [21]. Serial, anatomically matched cryosections from both control (n = 9) and stress (n = 9) animals were thaw-mounted on gelatin-coated slides from the level of the prefrontal cortex (bregma position 4.2 to 2.2) [23] and hippocampus (bregma position −2.8 to −4.3). Hippocampal cryosections were mounted in pairs (one control, one stress section per slide) and prefrontal cortical sections in groups of four (two control and two stress sections per slide). Individual slides thus held sections from each experimental group to minimize variations in hybridization conditions between experimental groups. Following hybridization (as described above), sections were coated with photoemulsion (Kodak NBT) at 42°C, dried for 90 min at RT, and stored for 7 weeks at 4°C in a light-proof container. Exposed slides were developed at 15°C for 5 min (Kodak developer D-19), rinsed twice briefly in H_2_0, fixed 5 min at RT (fixer, Kodak Polymax). Sections were counterstained with methyl-green (M-8884, Sigma), cleared in xylol, and coverslipped with mounting medium (Eukitt, Kindler, Freiburg, Germany). Hybridized sections were visualized with a 40× objective (NA = 1.4; Zeiss, Jena, Germany) under a light microscope (Axioscope, Zeiss) and silver grain quantification was performed on a cell by cell basis using the silver grain count function of MCID Basic software (Imaging Research Inc., St. Catherines, Ontario, Canada). ROD (relative optical density) threshold intensities were optimized to exclusively detect exposed silver grains: background interference from methyl-green was eliminated by the introduction of a green filter during quantification. The number of pixels contained within an individual silver grain was determined and used in subsequent calculations to extrapolate the number of silver grains within the area of interest. Circular counting masks of 125 pixel diameter were used to estimate silver grain number in hippocampal region CA3 and in prefrontal pyramidal neurons, whereas a smaller counting mask of 100 pixel diameter corresponding approximately to the size of a granule neuron cell body was used in the dentate gyrus to account for the tight packing of neurons within the granule cell layer. Boundaries delineating cortical laminae and the sub-areas of the prefrontal cortex were determined according to the published anatomical findings of Gabbott et al. [24]. Silver grain estimates were calculated from 2 sections per animal and 100 neurons per section within the dentate gyrus, CA3 pyramidal cell layer, anterior cingulate cortex, prelimbic cortex, and infralimbic cortex, respectively. For statistical analysis, the mean number of silver grains/brain area/rat was calculated and the individual data from stressed animals and controls were compared with the Student's t-test. Differences were regarded significant at p≤0.05.

### Immunocytochemistry for light microscopy

Animals received a lethal dose of ketamine, 50 mg/ml; xylazine, 10 mg/ml; atropine, 0.1 mg/ml) and were transcardially perfused first with saline (0.9% NaCl, for 2 min) and then with 4% paraformaldehyde in PBS (pH 7.2; for 10 min). Brains were removed, washed overnight in PBS and immersed in cryoprotectant (2% DMSO, 20% glycerol in 0.125 M PBS, pH 7.2) until saturation. Coronal cryosections (40 µm) were collected through prefrontal and hippocampal regions, washed briefly in PBS and quenched of endogenous peroxidase activity by 30 min incubation at room temperature (RT) in 0.5% H_2_0_2_ in distilled water. Sections were washed in 0.5% Triton X-100 (TX-100) in PBS, blocked for 1 hr at RT (5% normal rabbit serum and 0.5% TX-100 in PBS), incubated 48 hr at 4°C with monoclonal anti-M6a rat IgG (Medical & Biological Laboratories Co., Ltd, Japan; 1∶1000 dilution in 1% normal rabbit serum and 0.5% TX-100 in PBS), and washed again. Sections were then incubated in blocking solution (5% normal rabbit serum and 0.5% TX-100 in PBS) for 1 hr at RT, incubated with biotin-conjugated rabbit anti-rat IgG (DAKO, Hamburg, Germany; 1∶400 dilution in 1% normal rabbit serum and 0.5% TX-100 in PBS) for 4 hr at RT, then washed overnight at 4°C. The sections were treated with streptavidin-HRP (DAKO; 1∶200 dilution in 1% normal rabbit serum and 0.5% TX-100 in PBS) for 2 hr at RT, washed in PBS and then again in 0.05 M Tris/HCl (pH 7.2) prior to DAB detection (DAB detection was performed according to the manufacturer's instructions; DAB-Kit, Vector Laboratories, USA). Sections were washed in 0.05 M Tris/HCl (pH 7.6) and again in 0.1 M PBS prior to xylol clearance, dehydration, and coverslipping with Eukitt mounting medium (Kindler).

### Immunofluorescence and confocal microscopy

Antibodies used in double-labelling experiments were applied sequentially and blocking steps were performed using normal serum of host species from which respective secondary antibodies were derived. Cryostat sections (40 µm) from prefrontal cortex and hippocampus were rinsed in normal PBS and non-specific antibody binding sites were blocked with 3% normal serum, 0.3% TX-100 in PBS, for 1 hr at 4°C. Sections were then incubated in rat monoclonal anti-M6a (1/1500; in 3% normal serum, and 0.3% TX-100 in PBS) for 24 hr at 4°C, washed, and incubated in secondary antiserum (Alexa 594-coupled donkey anti-rat (Molecular Probes, Invitrogen, Leiden, the Netherlands) dilution 1/300 for 2 hr in a light proof container. Sections were washed and incubated in either rabbit anti-synaptophysin (Synaptic Systems, Göttingen, Germany), dilution 1/1000, or in mouse monoclonal anti microtubule-associated protein (MAP-2; Sigma), dilution 1/2000 in 3% normal serum, 0.5% TX-100 in PBS over night. Sections were then washed and incubated 2 hr at 4°C in secondary antiserum diluted 1/300 in 0.5% TX-100 in PBS: Alexa 488-coupled goat anti-rabbit IgG or Alexa 488-coupled goat anti-mouse IgG (Molecular Probes), respectively. Thereafter, sections were washed in PBS and floated/mounted on Histobond slides in PBS, allowed to dry overnight at 4°C and coverslipped with mounting medium (DakoCytomation, DAKO, Glostrup, Denmark).

Confocal microscopy was performed with a laser scanning microscope (LSM 5 Pascal, Zeiss, Göttingen, Germany) with an argon 488 nm and a helium/neon 543 nm laser. Analysis was performed in multiple tracking mode to avoid bleed-through between channels. The 543-nm laser was always used with a smaller detection pinhole diameter than the 488-nm laser to obtain the same optical slice thickness (slice thickness typically between 0.5–1.0 µm). High magnification images were obtained with an Apochromat 63× oil objective (NA = 1.4) and immersion oil (Immersol, Zeiss; refractive index = 1.518).

### Chronic restraint stress

For the experiment, male rats were housed individually in separate cages. Animals were randomly divided into two groups (n = 9/group) and allowed to habituate to the housing conditions and to daily handling for 10 days prior to the onset of experimentation. To expose rats to stress, we used a modified protocol of an established restraint stress paradigm [12], [13]. Accordingly, animals of the ‘Stress’ group were restrained daily for six hours (from 10:00 to 16:00, that is during the dark phase) for a total of 21 days in well-ventilated polypropylene tubes without access to food and water. Food was withheld from control animals during the restraint period to ensure that any effect on body weight gain was not simply a result of limited food availability. During restraint, animals were not physically compressed and did not experience pain. Bodyweights were recorded daily prior to the onset and during the entire period of daily restraint. For statistical evaluation, a day-by-day comparison of body weights was performed with paired t-tests using GraphPad Prism 4.03 (GraphPad Software, Inc., La Jolla, CA, USA). Differences were regarded significant at p≤0.05.

At the end of the experiment, 24 hrs following the last restraint, all animals were weighed and subsequently sacrificed. Brains were quickly dissected and adrenal glands were removed and weighed for analysis of relative adrenal weight.

## Results

### M6a splice variants Ia and Ib

A comparative real-time RT-PCR analysis of M6a transcript expression was performed in the brain and kidneys using primers specific for M6a isoforms Ia and Ib, and for the 3′-UTR region of the M6a transcript which is common to both isoforms (Fig. 1A). The results indicate that N-terminus variants of M6a are differentially expressed in central and peripheral tissues. M6a isoform Ia was found to be ubiquitously expressed at a low level in both brain and kidney, whereas variant Ib was identified as the predominant isoform expressed in the brain, especially in the hippocampal formation (Fig. 1B).

### M6a expression in hippocampal formation and prefrontal cortex

**Figure 2:**
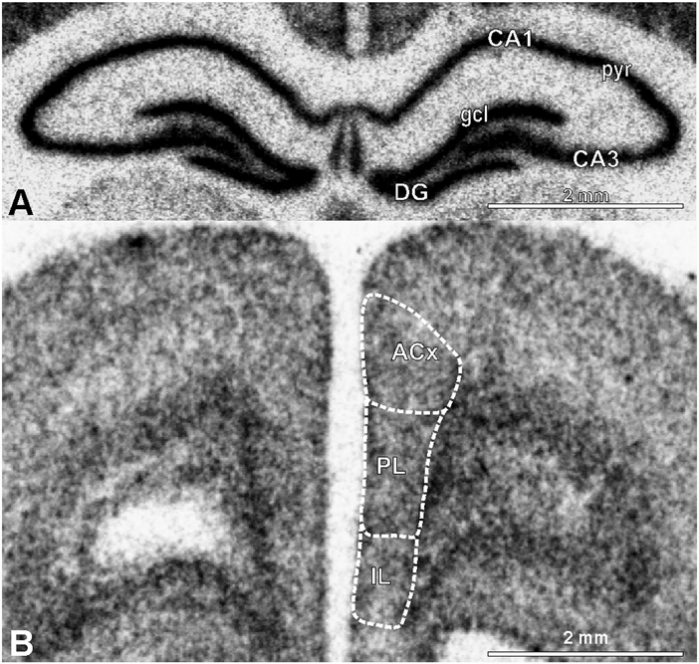
Autoradiograms showing M6a expression in the hippocampal formation (A) and the prefrontal cortex (B) as revealed by in situ hybridization. Abbreviations: ACx, anterior cingulated cortex; CA1, hippocampal region CA1; CA3, hippocampal region CA3; CA4, hippocampal region CA4; DG, dentate gyrus; gcl, granule cell layer; IL, infralimbic cortex; PL, prelimbic cortex; pyr, pyramidal cell layer.

**Figure 3:**
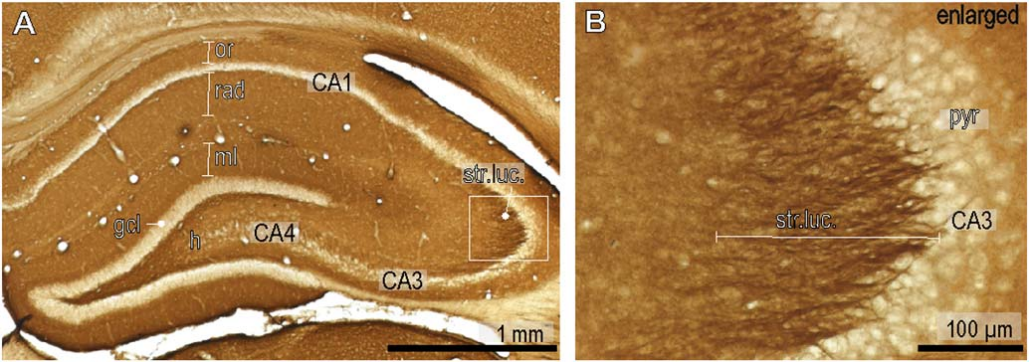
Immunocytochemical detection of M6a expression in the hippocampus. (A) shows no immunoreactivity in the granule cell layer (gcl) whereas the hilus (h) is strongly stained. A laminated pattern of immunoreactivity is detected in the molecular layer (ml) of the dentate gyrus, in stratum radiatum (rad) and stratum oriens (or) of region CA1. B (enlarged area from the box in A), mossy fibers terminating in the stratum lucidum (str.luc.) are strongly labeled by the M6a antibody whereas pyramidal neurons (pyr) are not stained.

**Figure 4:**
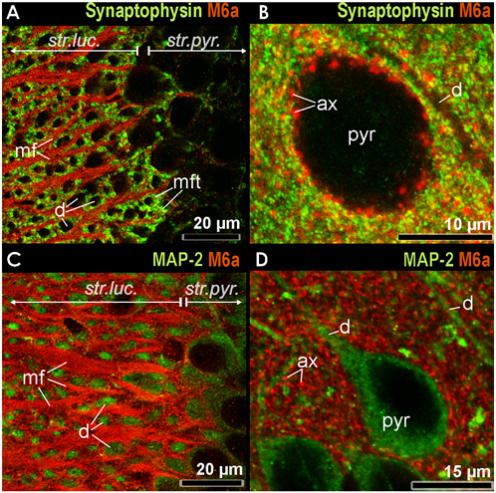
Immunofluorescence showing M6a immunoreactivity in the stratum lucidum (str.luc.) of the hippocampus (A, C) and in the infralimbic cortex (B, D) as revealed by confocal microscopy. A: M6a (red) and synaptophysin immunoreactivity (green) in the stratum lucidum. Note that the giant mossy fiber terminals (mft) which are strongly synaptophysin positive surround the unlabeled dendrites (d) of the CA3 pyramidal neurons. B: M6a (red) and synaptophysin immunoreactivity (green) in the infralimbic cortex; axons (ax) are M6a immunopositive, dendrites (d) are unlabeled. Note that there is occasional colocalization (yellow) of M6a and synaptophysin in the axonal terminals that surround the unlabeled soma of the pyramidal neuron (pyr). C: M6a (red) and MAP-2 immunoreactivity (green) in the stratum lucidum. Note that the dendrites (d) of CA3 pyramidal neurons which are strongly MAP-2 positive are not labelled by the M6a antibody. D: M6a (red) and MAP-2 immunoreactivity (green) in the infralimbic cortex. Note that there is no colocalization of M6a and MAP-2 in the axons/axonal terminals (ax) that surround the MAP-2 immunopositive soma of the pyramidal neurons (pyr).

We visualized M6a mRNA expression in the hippocampal formation and in the medial prefrontal cortex (mPFC) using in situ hybridization. The gene is strongly expressed in the pyramidal neurons of all hippocampal subfields (CA1–CA4) and in the granule cells of the dentate gyrus (Fig. 2a). Whereas M6a mRNA is concentrated in the cell bodies of the principal neurons, M6a protein is found in processes of those cells. Immunocytochemistry reveals that all hippocampal layers containing dense fiber networks are stained (Fig. 3). Strong M6a immunoreactivity is especially found in the stratum lucidum, the area where mossy fibers originating from the dentate gyrus granule neurons synapse on dendrites of CA3 pyramidal neurons (Fig. 3B). Immunofluorescence reveals that the membrane protein is concentrated in the mossy fiber axons (Fig. 4A). The giant mossy fiber terminals of these glutamatergic axons are strongly stained with the antibody against the synaptic vesicle protein synaptophysin. Co-staining with MAP-2 antibody which labels neuronal dendrites and cell bodies of pyramidal neurons reveals that M6a is not present in dendrites and cell bodies (Fig. 4C).

Moderate M6a mRNA expression is found in the three mPFC sub-areas, anterior cingulate, prelimbic and infralimbic cortex (Fig. 2B). In the mPFC, M6a immunoreactivity of cross cut axons appears as puncta which surround the somata of pyramidal neurons that are not stained with the synaptophysin antibody (Fig. 4B) but with MAP-2 antibody (Fig. 4D). These data confirm our previous results showing that M6a is a component of the membrane of glutamatergic axons but not of dendrites [6].

### Physiological effects of chronic stress

**Figure 5:**
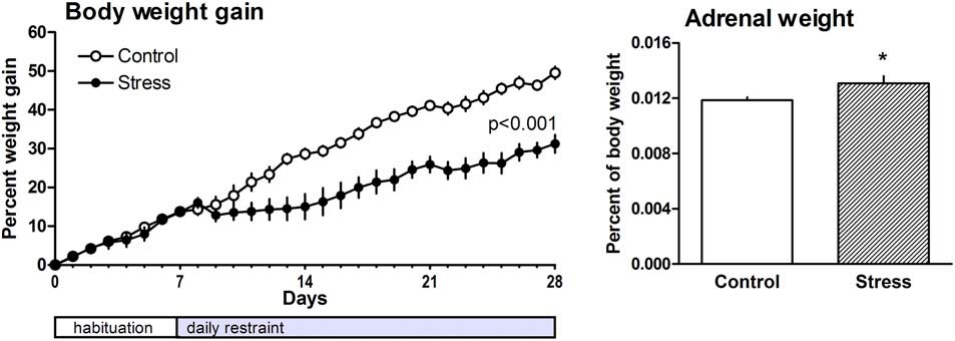
Effects of chronic restraint stress on body weight gain and adrenal weight. Left: Animals exposed to chronic restraint stress exhibited reduced body weight gain coinciding with the onset of restraint; data are from 11 controls and 10 stressed animals. Right: Adrenal weight of stressed rats is significantly increased compared to controls (10 animals/group). Data are expressed as mean±SEM (standard error of the mean). Significant differences between groups as determined by Student's *t*-test: *, p<0.05.

Coinciding with what has been shown previously [19] chronic restraint stress reduces body weight in male rats. Body weight gain differed significantly between rats submitted to daily restraint stress and controls (p<0.001, Student's t-test; Fig. 5A). At the end of the 3 weeks period of daily immobilization, the weight of adrenal glands relative to body weight was significantly increased compared to controls reflecting enhanced activity of the hypothalamus-pituitary-adrenal axis in stressed animals (Fig. 5B).

### M6a transcript expression after chronic stress

**Figure 6:**
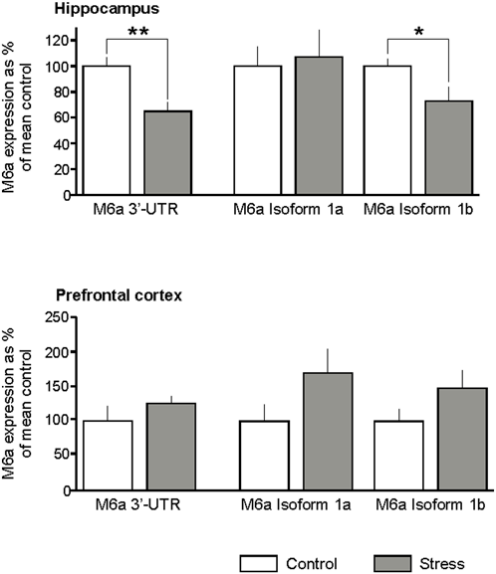
Quantitative real-time PCR showing M6a expression in the hippocampus (upper pannel) and prefrontal cortex (lower pannel). Analysis with primers recognizing the 3′-UTR of M6a, which is common to all isoforms of M6a, revealed a significant stress-induced downregulation in the hippocampus. Subsequent analysis with isoform-specific primers showed that M6a isoform Ib, but not Ia, is regulated by stress. In the prefrontal cortex, both isoforms show a tendency towards upregulation but fail to reach significance. Data are expressed as percentage of the mean control±SEM (standard error of the mean), n = 9/group. Significant differences between groups as determined by Student's *t*-test: *, p<0.05, **, p<0.01. UTR, untranslated region.

The effect of 21 days chronic restraint stress on M6a expression in specific brain regions was quantified with real-time PCR (Fig. 6). M6a 3′-UTR primers revealed a significant down-regulation of M6a transcripts (65% of controls, p<0.01) in the hippocampus of stressed animals. Subsequent analyses with isoform-specific primers demonstrated that isoform Ib (73% of controls, p<0.05), but not isoform Ia, is significantly reduced by stress in the hippocampal formation. RT-PCR detected no significant effect of stress on M6a expression in the prefrontal cortex, however, both isoforms Ia and Ib showed a tendency towards upregulation by stress, but failed to reach significance.

**Figure 7:**
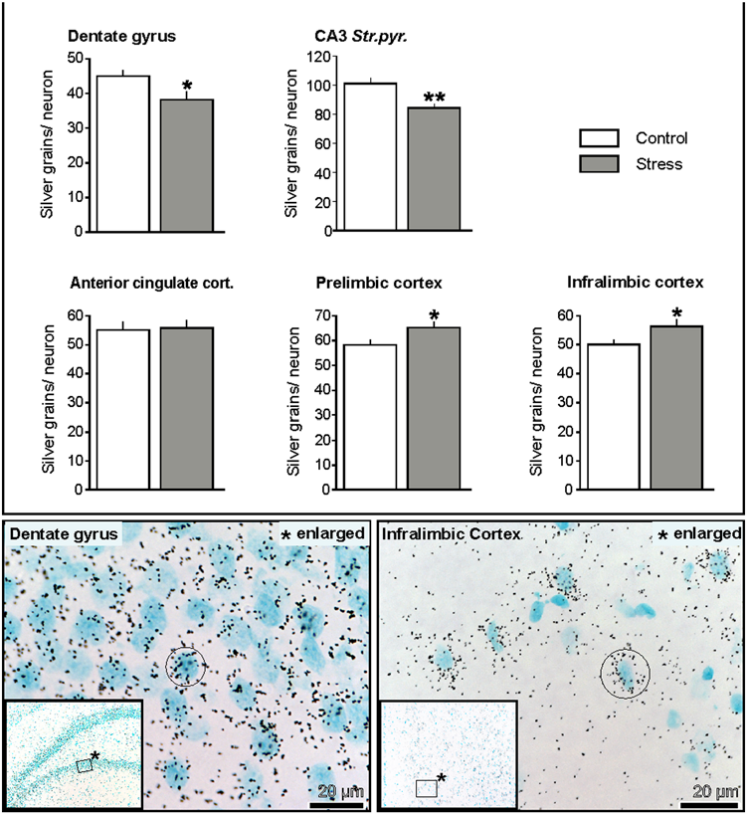
M6a expression in neurons of the hippocampal formation and the mPFC; quantitative in situ hybridization with emulsion autoradiography. Upper panel: Numbers of silver grains per neuron reveal reduced M6a mRNA expression after stress in dentate gyrus granule neurons and in CA3 pyramidal neurons, but enhanced M6a mRNA expression in neurons of the prelimbic and infralimbic cortex. Lower panel: Examples of sections from the dentate gyrus (left) and the infralimbic cortex (right) showing silver grains over cells that were counter stained with methyl-green (cyan). Significant differences between groups as determined by Student's *t*-test: *, p<0.05, **, p<0.01.

Quantitative *in situ* hybridization was performed to investigate the effects of chronic restraint stress on M6a expression in neurons of the hippocampal subfields and of the prefrontal cortex. Hybridization signals represented by silver grains appear as black puncta clustered over cells which were counterstained with methyl-green appearing blue (Fig. 7, bottom). M6a expression in granule neurons of dentate gyrus and CA3 pyramidal neurons was reduced to 85% (p<0.05) of controls. No effect of stress on M6a expression was detected in the anterior cingulate cortex, however, significant increases to approximately 112% (p<0.05) of controls were detected in the prelimbic and infralimbic cortex (Fig. 7).

## Discussion

It has been shown in the past that stress alters the structural organization of dendrites and of synapses on pyramidal neurons. Moreover, it has been concluded that such stress-induced changes would affect information transfer between the cells that communicate via axo-dendritic synapses [25]. The present data show that also axons are affected by stress. Daily restraint stress for three weeks reduces M6a expression in glutamatergic neurons of the hippocampal formation and may thus affect the structural integrity of the axons of those neurons. Since projections from CA3 hippocampal pyramidal neurons comprise a subset of axonal inputs to nuclei within the medial prefrontal cortex [26]–[27] our findings indicate that stress may affect communication between brain regions.

### M6a isoforms and axonal localization

The proteolipids including M6a, M6b, and PLP (major myelin proteolipid protein) are among the most abundantly expressed genes in the brain [28], [29]. The present quantitative RT-PCR analysis reveals that N-terminus variants of M6a are constitutively expressed at different levels within central and peripheral tissue: M6a isoform Ia is expressed at low levels in brain and kidney epithelia, whereas isoform Ib is highly expressed in the brain, but at very low levels in the kidneys. Previous studies addressing the function of M6a have suggested a role in ion transport, a hypothesis based initially on the immunolocalization of M6a to neuronal plasma membranes and the apical surface of polarized epithelia, both of which rely heavily on the coordinated transport of ions across membranes [4]. The findings of the present study reveal that distinct isoforms of M6a are differentially localised to neuronal and epithelial membranes, suggesting that splice variants of M6a may serve different functions in central and peripheral tissues.

We have previously shown by immunocytochemistry that M6a is present in axons of glutamatergic neurons with the strongest immunoreactivity being detected within the hippocampal formation [6]. The present data further confirm this: the membrane glycoprotein is located in the mossy fibers that originate in the dentate gyrus granule neurons and synapse on dendrites of CA3 pyramidal neurons. The giant axon terminals themselves are largely not labelled by the M6a antibody but are strongly stained by the antibody that binds to the synaptic vesicle marker protein synaptophysin. Colocalization is only observed as a result of close proximity between synaptophysin-immunoreactive vesicles and M6a as a component of the terminal membrane.

Hippocampal pyramidal neurons were also found to express M6a, with no apparent difference in expression levels observed between subfields of the cornu ammonis (CA). Axonal projections from CA3 pyramidal neurons within the hippocampal formation include Schaffer collaterals terminating on the dendrites of CA1 pyramidal neurons within stratum radiatum, and associational projections terminating on the apical dendrites of CA3 pyramidal neurons within stratum radiatum. Schaffer collaterals diverge extensively throughout the longitudinal axis of the hippocampal formation [30] and are therefore not visualized as a coherent fiber pathway. Instead, M6a targeted to the terminal regions of CA3 projections is primarily detected as synaptic puncta within the stratum radiatum.

### M6a is downregulated by chronic stress in the hippocampus

As determined by quantitative real-time RT-PCR chronic restraint selectively downregulates neuronally expressed M6a isoform Ib in the hippocampus, but not isoform Ia. M6a was initially identified by subtractive hybridization as a glucocorticoid responsive gene in tree shrews chronically treated with cortisol [7], suggesting stress-induced reductions in hippocampal M6a expression may occur via glucocorticoid-regulated repression of transcription [31]. Downregulation of M6a mRNA in the hippocampal formation of chronically restrained rats is consistent with previous data demonstrating reduced M6a expression in the hippocampus of chronically restrained mice [8]. Moreover, M6a mRNA was also downregulated in the hippocampal formation of psychosocially stressed tree shrews [11] indicating that the effect of stress on M6a expression is robustly conserved across species and is reproducible with different stress paradigms.

### M6a in the prefrontal cortex

The medial prefrontal cortex comprises functionally distinct sub-areas of which the PL and IL are particularly involved in the integration of autonomic and cognitive information ultimately contributing to the perception of stress [32]–[37]. In situ hybridization with emulsion autoradiography showed that M6a is abundantly expressed in all mPFC layers in large neurons bearing the morphological characteristics of pyramidal neurons. Moreover, pyramidal neurons within layers II/III exhibited comparable levels of expression in all sub-areas examined, ACx, PL and IL, as determined by quantitative in situ hybridization. PFC pyramidal neurons receive synaptic inputs in an organized fashion: Distal portions of the apical dendritic tree (cortical layer I) receive inputs primarily from extracortical regions, such as the medial dorsal thalamic nuclei and hippocampal CA3 subfield [38], [39], whereas proximal portions of apical and basilar dendrites receive inputs primarily from local cortical circuits [40].

Quantitative RT-PCR permits the detection of changes in gene expression with high sensitivity, however, the anatomical specificity of data generated with this method relies on the ability to precisely excise the tissue/cells of interest. As described, we detected a tendency towards increased M6a expression in chronically restrained rats, but this tendency failed to reach significance. Since significant changes in gene expression within the mPFC may be masked in a combined analysis of all sub-areas, in situ hybridization was performed which allows a semi-quantitative evaluation of mRNA transcript abundance in single neurons. From a methodological perspective, in situ hybridization demonstrated less sensitivity to stress-induced changes in hippocampal M6a expression compared to quantitative RT-PCR analyses, but enabled expression to be quantified within specific neurons. In the mPFC, three weeks restraint increased M6a expression in pyramidal neurons (layers II/III) of PL and IL whereas no change of expression was observed in the ACx.

In previous studies, dendritic remodelling observed in pyramidal neurons within layers II/III of the mPFC was interpreted to represent an adaptive response to altered synaptic input from extracortical sources such as the CA3 region of the hippocampus [41]. It is conceivable that the increased M6a expression observed in pyramidal neurons of PL/IL reflects an adaptive mechanism designed to strengthen associational contacts and in doing so, to sensitize pyramidal neurons to weakened inputs form the hippocampus.

### Possible implications of M6a regulation in glutamatergic axons

As shown in our previous study [6] M6a is present in axonal membranes and may as such play an important role in the structural integrity of axons. Since the membrane glycoprotein is in particular strongly expressed in the mossy fibers one has to assume that stress changes the integrity of those axonal projections from the granule cells to CA3 pyramidal neurons. Indeed, three weeks of daily restraint changed the morphology of the giant mossy fiber terminals in the stratum lucidum [25]. Maladaptive changes in mossy fiber terminal morphology induced by stress are likely to have a profound impact on transmission within the hippocampal circuits and may contribute to perturbations in glutamatergic transmission previously associated with chronic stress [42]–[43]. Moreover, also stress-induced changes in other hippocampal subregions such as CA1 may be related to reduced M6a expression [44]. Altogether, these changes may contribute to the inhibition of long-term potentiation that has been recorded in the hippocampus after stress [45]–[46].
